# Supplementary material for: Composite branched and linear F-actin maximize myosin-induced membrane shape changes in a biomimetic cell model
Source: Commun Biol. 2024 Jul 10;7:840. doi: 10.1038/s42003-024-06528-4 (PMC11236970; doi:10.1038/s42003-024-06528-4)
Supplement: Supplementary file 1 — Supplementary Information [file 42003_2024_6528_MOESM1_ESM.pdf]

## Supplementary Information for

# Composite branched and linear F-actin maximize myosin-induced membrane shape changes in a biomimetic cell model

Ryota Sakamoto and Michael P. Murrell*

*Corresponding author: **Email**: michael.murrell@yale.edu

**This PDF file includes:**

Supplementary Figures 1-11

Supplementary References 1-3

**Supplementary Figures**

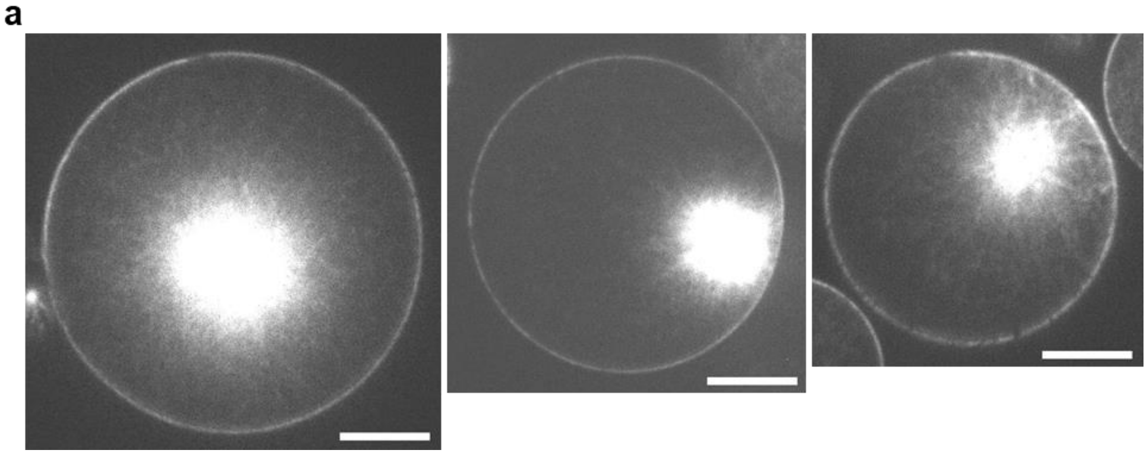

**Supplementary Figure 1. Actomyosin network contraction within liposomes in the absence of blebbistatin.** (**a**) Snapshots showing the liposomes containing actin and myosin without blebbistatin present after the preparation of liposomes. The actomyosin network had already contracted during the preparation of liposomes (~30 min). Scale bars, 10 µm.

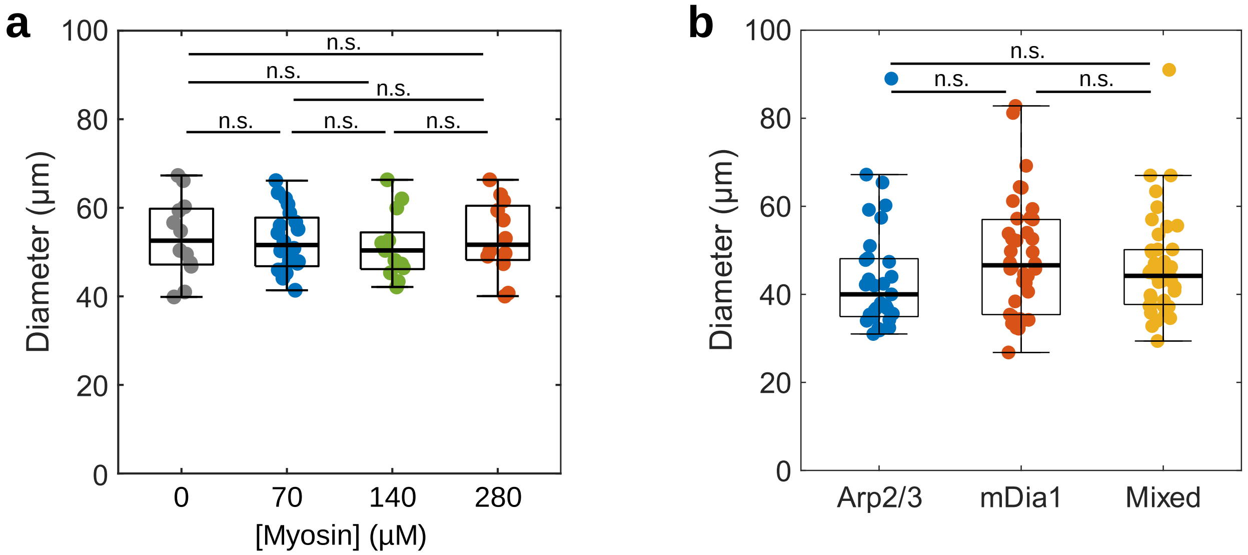


**Supplementary Figure 2. The analyzed liposome size was comparable in different conditions.** (**a**) Boxplot showing the liposome diameter analyzed for the volume network contraction at different myosin concentration (n=12 liposomes and N=2 independent experiments in 0 nM; n=20 and N=2 in 70 nM; n=13 and N=2 in 140 nM; n=12 and N=3 in 280 nM). (**b**) Boxplot showing the liposome diameter analyzed for liposome deformation in different F-actin cortex architectures (n=31 and N=6 in Arp2/3; n=38 and N=6 in mDia1; n=43 and N=11 in Mixed). n.s.: not significant.

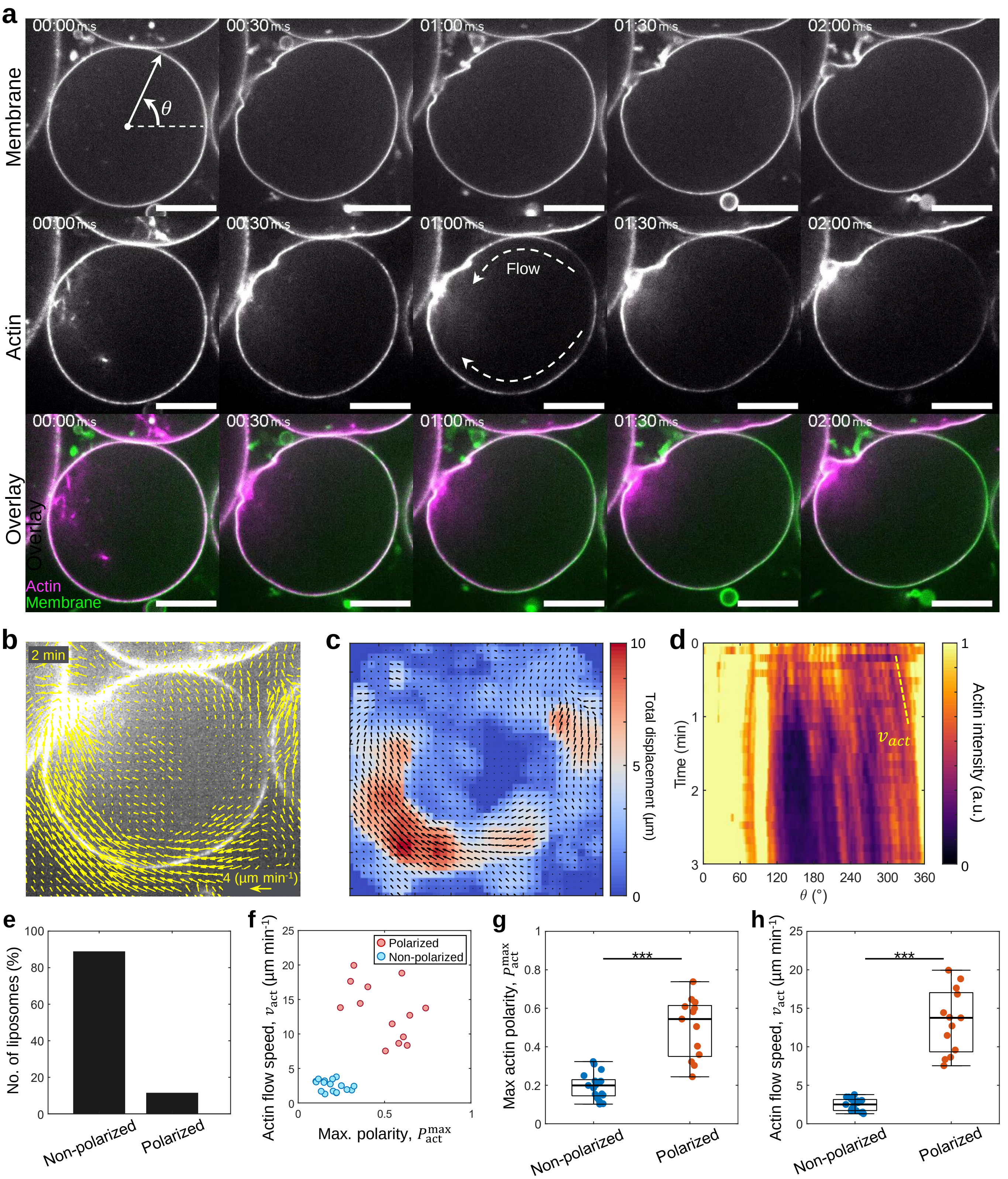


**Supplementary Figure 3. Polarized actin flow.** (**a**) Timelapse images showing the polarized actin flow in a mixed cortex liposome at [Arp2/3]:[mDia1]=1:1 (Movie S1). (**b**) Cortical actin flow. Yellow arrows are the PIV velocity vectors. (**c**) Total displacement field. Black arrows are the total displacement vectors over 3 min and vector magnitudes are normalized by its maximum. (**d**) Kymograph of actin fluorescence intensity extracted along the membrane in (a). Actin flow speed $v_{act}$ is analyzed along the dashed line. (**e**) Frequency of liposomes, calculated as the ratio of liposomes with actin flow compared to the total number of liposomes tested in each experiment (n=102 non-polarized and n=13 polarized, N=6 independent experiments). (**f**) Maximum polarity vs actin flow speed (n=17 and N=2 in non-polarized; n=13 and N=4 in polarized). (**g** and **h**) Boxplot showing the maximum actin polarity (g) and the actin flow speed (h). *** represents $p$<0.001. Scale bars, 10 μm.

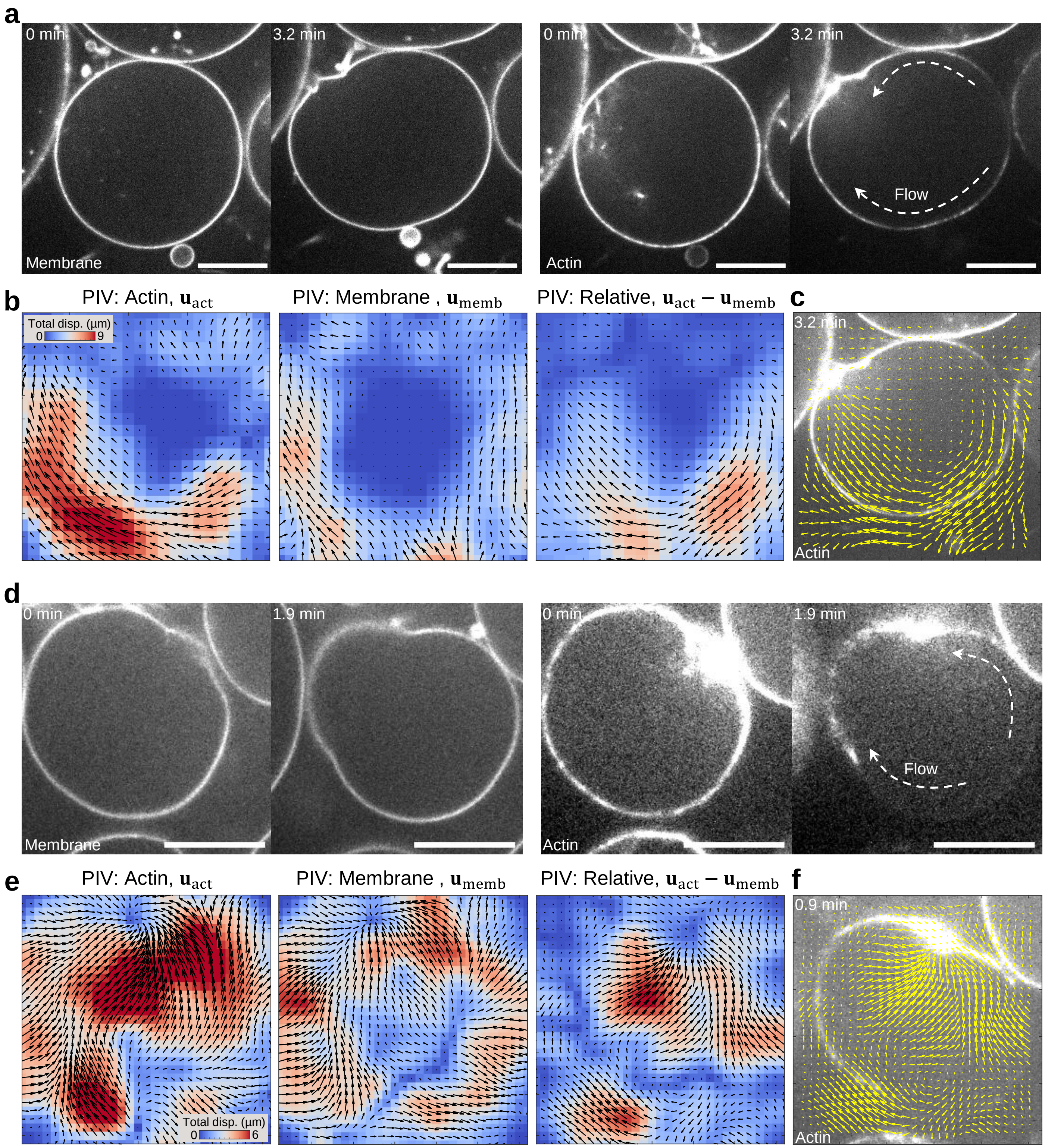


**Supplementary Figure 4. PIV of actin relative to membrane deformation.** The displacement/deformation of the membrane could affect the PIV of actin flows. To eliminate the influence of membrane deformation-induced displacement, we performed PIV analysis on the membrane channel, yielding $\mathbf{u}_{memb}$, which was subtracted from the PIV vectors of the actin fluorescence, $\mathbf{u}_{act}$. The resulting relative displacement field should reflect the displacement of actin flow alone. (**a** and **d**) Snapshots showing the membrane (left) and actin (right) of liposomes with polarized actin flow. (**b** and **e**) Total displacement field of actin (left), membrane (center), and actin relative to the membrane. Black arrows are the total displacement vectors, and vector magnitudes are normalized by their maximum. (**c** and **f**) Overlay of the relative displacement vectors on the actin channel. It should be noted that, although actin fluorescence is localized, the fluorescence intensity is slightly leaked/broadened around the center of the cortex. This results in slightly broadened PIV vectors around the cortex. Scale bars, 10 µm.

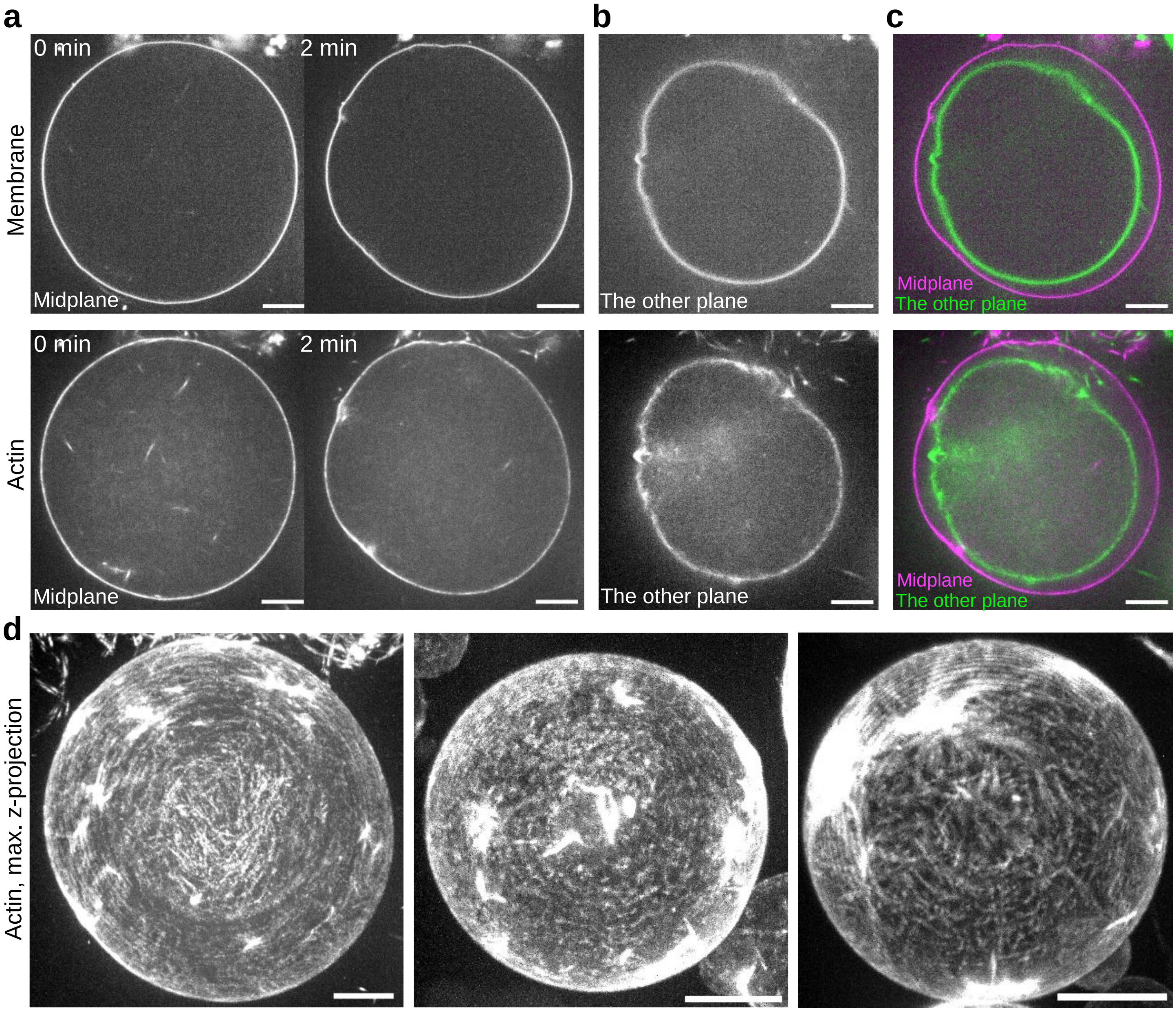


**Supplementary Figure 5. Membrane deformation outside of the midplane and z-projection images.** (**a**) Snapshots showing the light activation at the midplane of the mixed cortex architecture liposomes ([mDia1]:[Arp2/3]=1:1). (**b**) A snapshot showing the membrane deformation outside of the midplane after the light activation at the midplane in the same liposome as in (a). (**c**) Overlay of the midplane (magenta) and the outside of the midplane (green) in a post-deformed liposome. (**d**) Maximum projection of the z-stack images of the post-light activated liposomes in the actin fluorescence showing multiple aster-like structures across the surface of the liposomes. Scale bars, 10 μm.

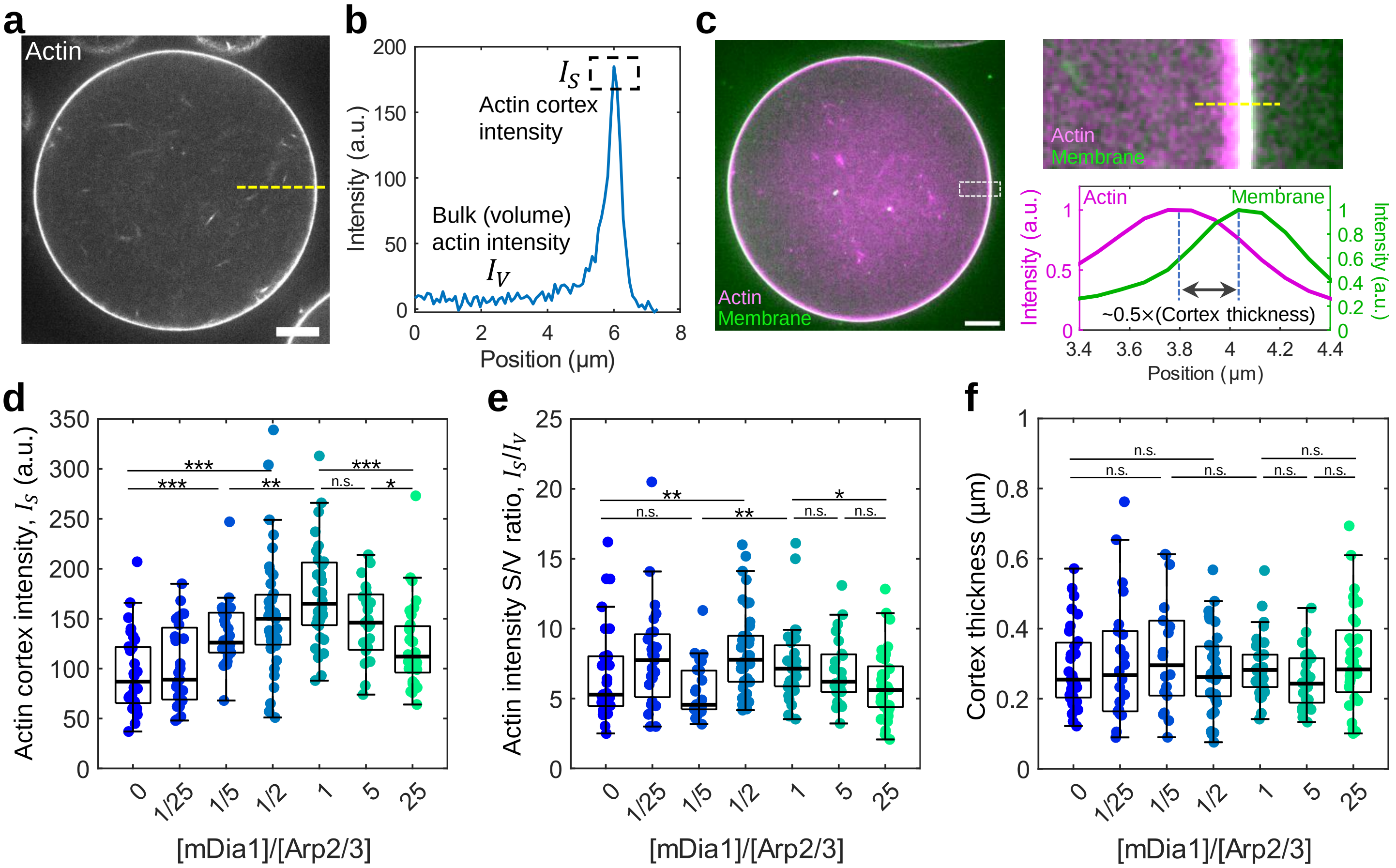


**Supplementary Figure 6. Cortex intensity and width measurement for varied mDia1 to Arp2/3 ratio.** (**a**) A snapshot showing the liposome at [mDia1]/[Arp2/3] = 1 before 405 nm laser illumination. (**b**) Spatial profile of the actin fluorescence intensity is extracted along the yellow dashed line in (a). Actin cortex intensity, $I_S$, is defined as a peak intensity, while bulk actin intensity, $I_V$, is defined as the intensity of the plateau region. The fluorescence intensities were extracted by fitting a gauss function, $y = a + (b - a)\exp[-(x - c)^2/2d^2]$, to the spatial profile of the actin intensity, where $a = I_V$, $b = I_S$. (**c**) A snapshot showing the overlay of the actin fluorescence and membrane fluorescence. A magnified image within the yellow dashed square is shown on the right. The spatial intensity profile along the dashed yellow line is shown. The cortex thickness was estimated from the distance between the peak of the actin fluorescence and the membrane fluorescence using the standard method [1,2]. Briefly, using the distance between the peaks of the membrane fluorescence and the actin cortex fluorescence, Δ, the cortex thickness, $h$, is ideally estimated as $h \simeq 2\Delta$. However, due to the presence of actin in the liposome volume, the position of the peak is slightly shifted to the liposome center by a distance $\delta$ [1]. The shift distance $\delta$ is given by $\delta = (\sigma^2/h)\ln[(I_{\mathrm{out}} - I_S)/(I_V - I_S)]$, where $I_{\mathrm{out}}$ is the intensity outside of the liposome, and the standard deviation, $\sigma \simeq 119$ nm, of the point spread function of the microscope is estimated by fitting a Gaussian function to the fluorescence of sub-resolution beads (Supplementary Fig. 5). Combining this with the relation, $h = 2(\Delta - \delta)$[1], the cortex thickness was estimated for each liposome. (**d**) Boxplot showing actin cortex intensity $I_S$ (n=32 liposomes and N=2 independent experiments in [mDia1]/[Arp2/3]=0; n=27 and N=2 in 1/25; n=26 and N=2 in 1/5; n=39 and N=2 in 1/2; n=31 and N=1 in 3; n=23 and N=2 in 5; n=33 and N=2 in 25). (**e**) Boxplot showing actin intensity surface to volume ratio (S/V ratio), $I_S/I_V$ (n=32 liposomes and N=2 independent experiments in [mDia1]/[Arp2/3]=0; n=27 and N=2 in 1/25; n=26 and N=2 in 1/5; n=39 and N=2 in 1/2; n=31 and N=1 in 3; n=23 and N=2 in 5; n=33 and N=2 in 25). (**f**) Boxplot showing cortex thickness (n=31 liposomes and N=2 independent experiments in [mDia1]/[Arp2/3]=0; n=22 and N=2 in 1/25; n=18 and N=2 in 1/5; n=33 and N=2 in 1/2; n=30 and N=1 in 3; n=22 and N=2 in 5; n=32 and N=2 in 25). *, **, *** represent $p<0.05$, $p<0.01$, and $p<0.001$, respectively. n.s., not significant. Scale bars, 10 μm.

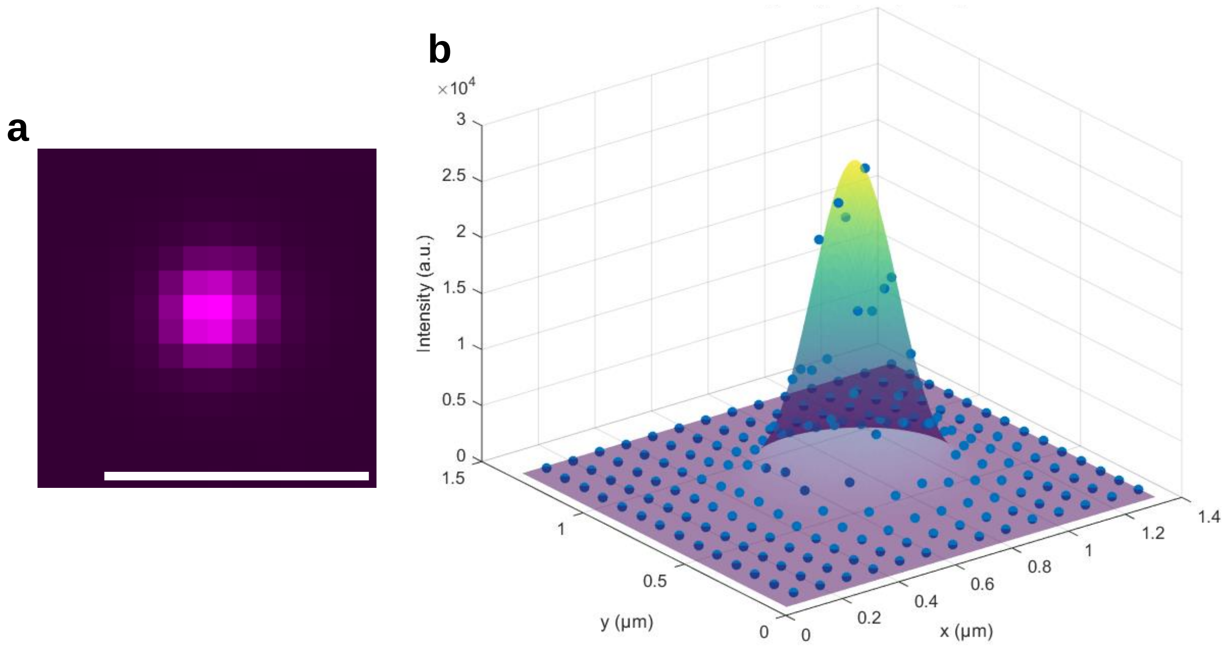


**Supplementary Figure 7. Point spreading function analysis.** (**a**) A snapshot showing the sub-resolution (diameter=100 nm) fluorescent beads in the 561 nm channel. (**b**) 3D plots showing the fitting of a Gaussian function (curved plane) to the pixel intensity points (spheres) to extract the standard deviation $\sigma$ of the point spreading function [1]. The intensity value of the curved plane is color-coded. The average $\sigma$ obtained from the fitting was $\sigma = 119 \pm 2$ nm (n=10 beads and N=2 independent experiments). Scale bar, 1 µm.

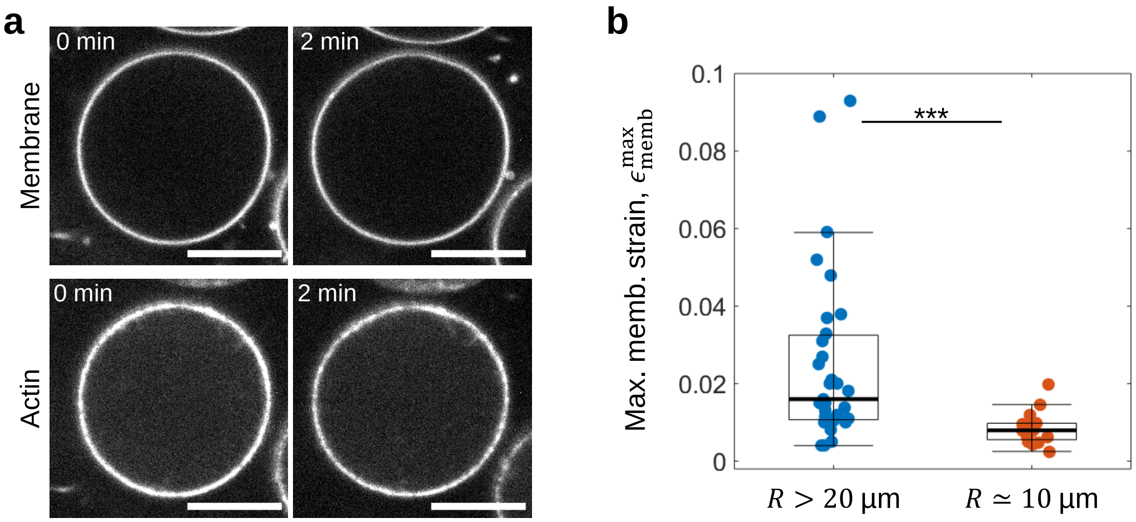


**Supplementary Figure 8. Membrane deformation of smaller liposomes.** (**a**) A snapshot showing the membrane deformation dynamics of a liposome with radius $R \simeq 10$ μm at [Arp2/3]:[mDia1]=1:1. (**b**) Boxplot showing maximum membrane strain comparing the liposomes for $R > 20$ μm and $R \simeq 10$ μm different His-VCA concentrations (n=31 liposomes and N=2 independent experiments in $R > 20$ μm; n=16 and N=2 in $R \simeq 10$ μm). The membrane strain for liposomes with $R \simeq 10$ μm was significantly smaller than that for liposomes with $R > 20$ μm. This behavior could be explained by the bending energy cost for the smaller liposomes being much larger than that for the larger ones. Based on the mechanical energy of deformation described in the main text, the ratio of the bending energy cost to the stretching energy cost of the actin cortex scales with radius (curvature), $F_{\mathrm{bend}}/F_{\mathrm{stretch}} \sim h^2 R^{-2}$, when the deformation size (correlation length) is comparable to the liposome size, $\xi_{\mathrm{c}} \sim R$ [3]. Thus, for the smaller liposomes, the bending energy cost dominates, making it difficult to deform the membrane. In addition to this theoretical consideration, experimentally, the smaller liposomes would have less membrane-localized myosin due to the larger surface-to-volume ratio, which decreases the net active stress applied to the actin cortex. Together, these contributions may limit the extent of deformation for the smaller liposomes. *** represents $p$<0.001. Scale bars, 10 μm.

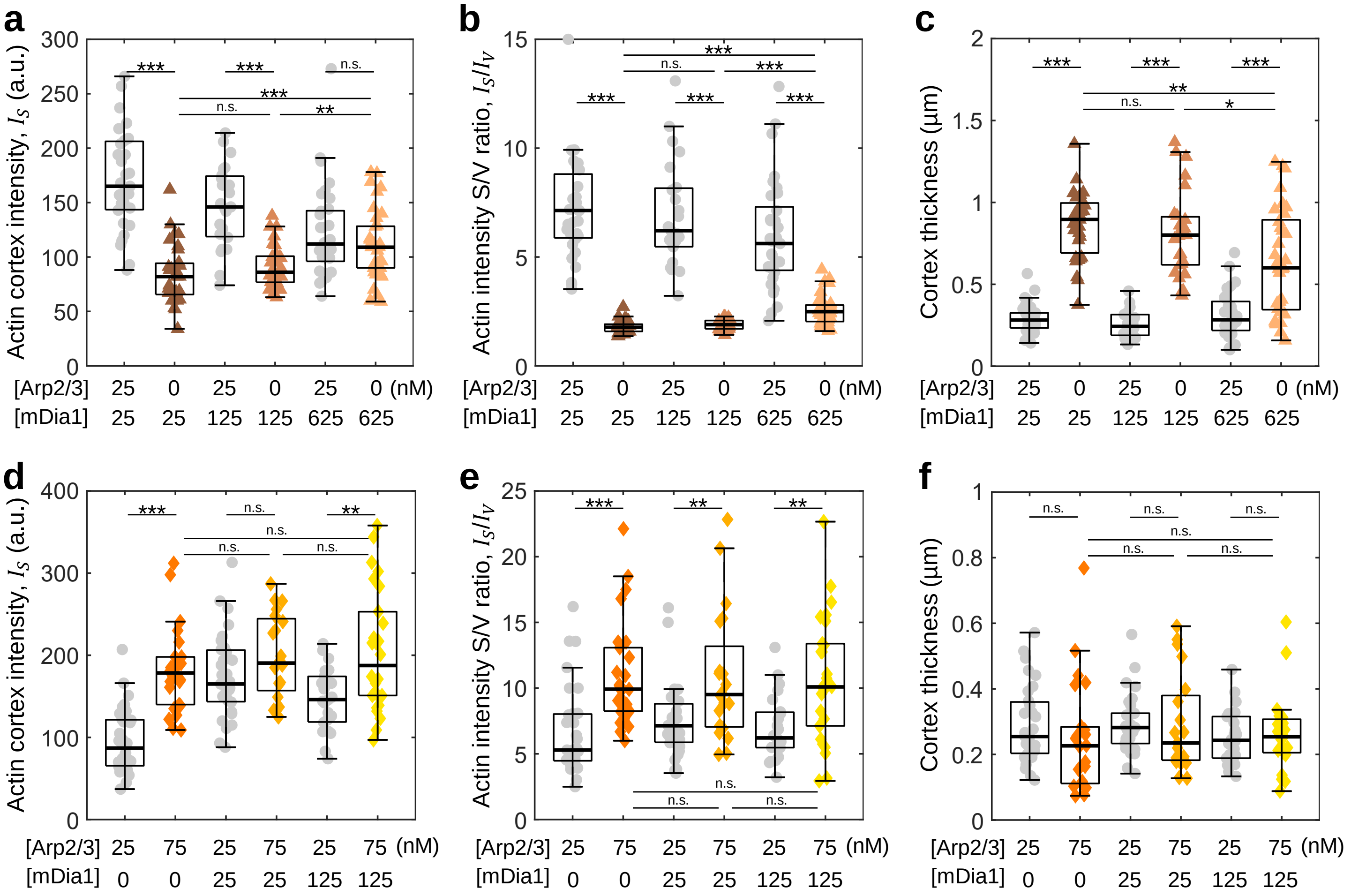


**Supplementary Figure 9. Cortex intensity and width measurement for liposomes with mDia1 only or liposomes with higher Arp2/3 concentration.** (**a**) Boxplot showing actin cortex intensity $I_S$ (n=31 liposomes and N=2 independent experiments in [mDia1]=25 nM; n=29 and N=2 in [mDia1]=125 nM; n=37 and N=2 in [mDia1]=625 nM). (**b**) Boxplot showing actin intensity surface to volume ratio (S/V ratio), $I_S/I_V$(n=31 liposomes and N=2 independent experiments in [mDia1]=25 nM; n=29 and N=2 in [mDia1]=125 nM; n=37 and N=2 in [mDia1]=625 nM). (**c**) Boxplot showing cortex width (n=31 liposomes and N=2 independent experiments in [mDia1]=0 nM; n=27 and N=2 in [mDia1]=25 nM; n=33 and N=2 in [mDia1]=125 nM). (**d**) Boxplot showing actin cortex intensity $I_S$ (n=26 liposomes and N=3 independent experiments in [mDia1]=0 nM; n=20 and N=2 in [mDia1]=25 nM; n=26 and N=3 in [mDia1]=125 nM). (**e**) Boxplot showing actin intensity surface to volume ratio (S/V ratio), $I_S/I_V$ (n=26 liposomes and N=3 independent experiments in [mDia1]=0 nM; n=20 and N=2 in [mDia1]=25 nM; n=26 and N=3 in [mDia1]=125 nM). (**f**) Boxplot showing cortex width (n=22 liposomes and N=3 independent experiments in [mDia1]=0 nM; n=20 and N=2 in [mDia1]=25 nM; n=22 and N=3 in [mDia1]=125 nM). *, **, *** represent $p$<0.05, $p$<0.01, and $p$<0.001, respectively. n.s., not significant.

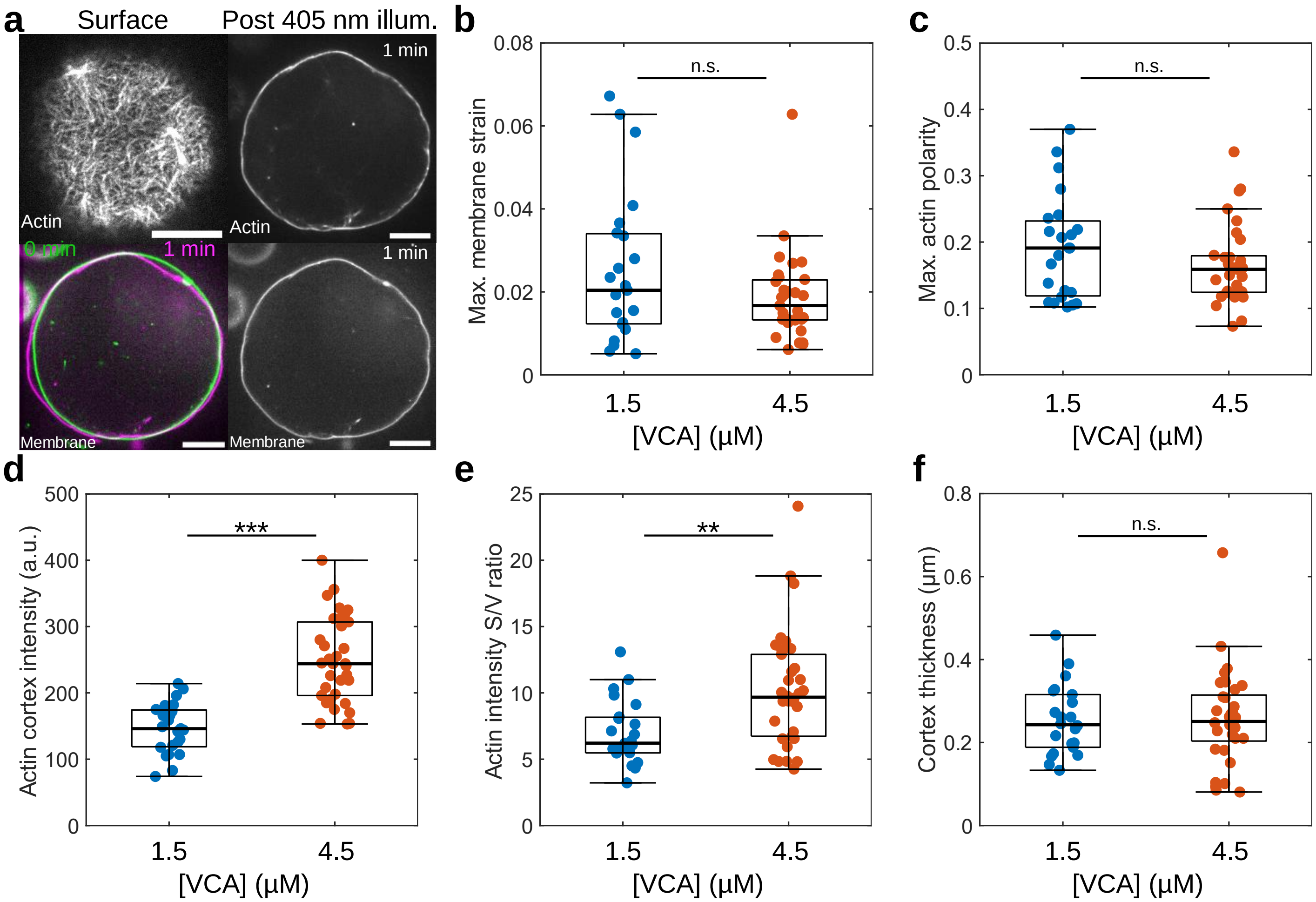


**Supplementary Figure 10. Membrane deformation and cortex intensity analysis on higher VCA concentration.** (**a**) A snapshot showing the actin cortex by focusing on the bottom surface of the liposome (left). Before (green) and post 405 nm illumination (magenta) membrane are overlayed (left). The right images showing the actin cortex and membrane with deformations 2 min after 405 nm laser illumination. The concentration of His-VCA is 4.5 μM and mDia1 to Arp2/3 ratio is [mDia1]/[Arp2/3] = 5. (**b**) Boxplot showing maximum membrane strain for different His-VCA concentrations (n=23 liposomes and N=2 independent experiments in [VCA]=1.5 μM; n=31 and N=2 in [VCA]=4.5 μM). (**c**) Boxplot showing maximum actin polarity (n=23 and N=2 in [VCA]=1.5 μM; n=31 and N=2 in [VCA]=4.5 μM). (**d**) Boxplot showing actin cortex intensity $I_S$ (n=23 and N=2 in [VCA]=1.5 μM; n=34 and N=2 in [VCA]=4.5 μM). (**e**) Boxplot showing actin intensity surface to volume ratio (S/V ratio), $I_S/I_V$ (n=23 and N=2 in [VCA]=1.5 μM; n=34 and N=2 in [VCA]=4.5 μM). (**f**) Boxplot showing cortex width (n=22 and N=2 in [VCA]=1.5 μM; n=33 and N=2 in [VCA]=4.5 μM). *, **, *** represent $p$<0.05, $p$<0.01, and $p$<0.001, respectively. n.s., not significant. Scale bars, 10 μm.

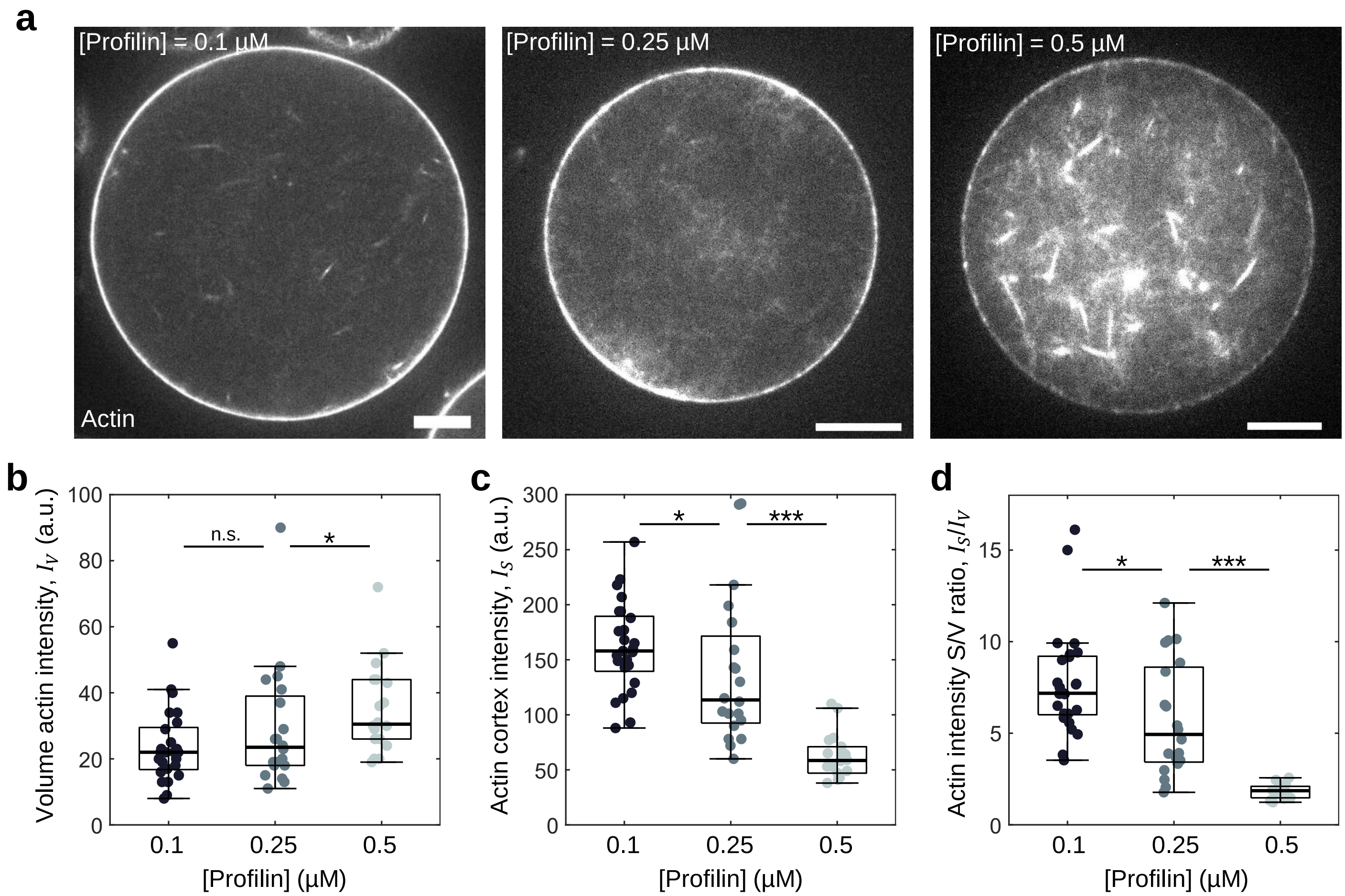


**Supplementary Figure 11. Actin cortex intensity analysis on varied profilin concentration.** (**a**) Snapshot showing the actin cortex liposome at [mDia1]/[Arp2/3] = 1 before 405 nm laser illumination with varied profilin concentration. (**b**) Boxplot showing volume actin intensity $I_V$ (n=25 liposomes and N=2 independent experiments in [Profilin]=0.1 μM; n=20 and N=2 in [Profilin]=0.25 μM; n=18 and N=2 in [Profilin]=0.5 μM). (**c**) Boxplot showing actin cortex intensity $I_S$. (**d**) Boxplot showing surface to volume ratio of the actin intensity, $I_S/I_V$. * and *** represent $p<0.05$ and $p<0.001$, respectively. n.s., not significant. Scale bars, 10 μm.

## Supplementary References

[1]. Clark, G., Dierkers, K., & Paluch, E. K. Monitoring actin cortex thickness in live cells. *Biophys. J.* **105**, 570-508 (2013).

[2]. Chugh, P., et al. Actin cortex architecture regulates cell surface tension. *Nat. Cell Biol.* **19**, 689-697 (2017).

[3]. Ito, H., Nishigami, Y., Sonobe, & S., Ichikawa, M. Wrinkling of a spherical lipid interface induced by actomyosin cortex. *Phys. Rev. E* **92**, 062711 (2015).
