# Supplementary material for: Composite branched and linear F-actin maximize myosin-induced membrane shape changes in a biomimetic cell model
Source: Commun Biol. 2024 Jul 10;7:840. doi: 10.1038/s42003-024-06528-4 (PMC11236970; doi:10.1038/s42003-024-06528-4)
Supplement: Supplementary file 2 — Description of Additional Supplementary Materials [file 42003_2024_6528_MOESM2_ESM.pdf]

Description of Additional Supplementary Files

File name: Supplementary Data 1

Description: The source data underlying the graphs in the paper

File name: Supplementary Movie 1

Description: Time-lapse movie of actomyosin contraction at myosin concentrations of 280 nM, 140 nM, 70 nM, and 0 nM in Fig. 1. The liposomes were kept in the dark for more than 10 minutes after preparation. Subsequently, the 405 nm laser was shined at each frame. Scale bar is 10 μm

File name: Supplementary Movie 2

Description: Time-lapse movie of a liposome with the Arp2/3-nucleated cortex in Fig. 2. The liposomes were kept in the dark for more than 10 minutes after preparation. Subsequently, the 405 nm laser was shined at each frame. Arp2/3 and His-VCA concentration is 25 nM and 1.5 μM, respectively. Membrane (green) and actin (magenta) were imaged. Scale bar is 10 μm.

File name: Supplementary Movie 3

Description: Time-lapse movie of a liposome with the Arp2/3-nucleated cortex. The liposomes were kept in the dark for more than 10 minutes after preparation. Subsequently, the 405 nm laser was shined at each frame. Arp2/3 and His-VCA concentration is 25 nM and 1.5 μM, respectively. Membrane (green) and actin (magenta) were imaged. Scale bar is 10 μm.

File name: Supplementary Movie 4

Description: Time-lapse movie of a liposome with the mDia1-nucleated cortex in Fig. 2. The liposomes were kept in the dark for more than 10 minutes after preparation. Subsequently, the 405 nm laser was shined at each frame. mDia1 concentration is 25 nM. Profilin concentration is 0.1 μM. Membrane (green) and actin (magenta) were imaged. Scale bar is 10 μm.

File name: Supplementary Movie 5

Description: Time-lapse movie of a liposome with the mDia1-nucleated cortex. The liposomes were kept in the dark for more than 10 minutes after preparation. Subsequently, the 405 nm laser was shined at each frame. mDia1 concentration is 25 nM. Profilin concentration is 0.1 μM. Membrane (green) and actin (magenta) were imaged. Scale bar is 10 μm.

File name: Supplementary Movie 6

Description: Time-lapse movie of a liposome with the mixed architecture cortex in Fig. 2. The liposomes were kept in the dark for more than 10 minutes after preparation. Subsequently, the 405 nm laser was shined at each frame. Arp2/3 and His-VCA concentration is 25 nM and 1.5 μM, respectively. mDia1 concentration is 25 nM. Profilin concentration is 0.1 μM. Membrane (green) and actin (magenta) were imaged. Scale bar is 10 μm.

File name: Supplementary Movie 7

Description: Time-lapse movie of a liposome with the mixed architecture cortex. The liposomes were kept in the dark for more than 10 minutes after preparation. Subsequently, the 405 nm laser was shined at each frame. Arp2/3 and His-VCA concentration is 25 nM and 1.5 μM, respectively. mDia1 concentration is 25 nM. Profilin concentration is 0.1 μM. Membrane (green) and actin (magenta) were imaged. Scale bar is 10 μm.

File name: Supplementary Movie 8

Description: Time-lapse movie of a liposome with the mixed architecture cortex showing cortical actin flow. The liposomes were kept in the dark for more than 10 minutes after preparation. Subsequently, the 405 nm laser was shined at each frame. Arp2/3 and His-VCA concentration is 1.5 μM, respectively. mDia1 concentration is 25 nM. Profilin concentration is 0.1 μM. Membrane (green) and actin (magenta) were imaged. Scale bar is 10 μm.

File name: Supplementary Movie 9

Description: Time-lapse movie of a liposome with the mixed architecture cortex showing cortical actin flow. The liposomes were kept in the dark for more than 10 minutes after preparation. Subsequently, the 405 nm laser was shined at each frame. Arp2/3 and His-VCA concentration is 1.5 μM, respectively. mDia1 concentration is 25 nM. Profilin concentration is 0.1 μM. Membrane (green) and actin (magenta) were imaged. Scale bar is 10 μm.
